# Single-fraction high-dose-rate brachytherapy as monotherapy for localized prostate cancer: long-term follow-up study based on meta-analysis

**DOI:** 10.7150/jca.104279

**Published:** 2025-01-01

**Authors:** Li Xiao, Li-Li Yu, Li-Yuan Zhang, Wei Guo, Li-Xin Liu, Yun-Chuan Sun, Xuan Kan, Kai Zhang

**Affiliations:** 1 Department of Radiation Oncology, Hebei Province Cangzhou Hospital of Integrated Traditional and Western Medicine, Cangzhou, Hebei, 061000, China.; 2 Hebei Province Integrated Traditional Chinese and Western Medicine 3D Printing Technology Innovation Center, Cangzhou, Hebei, 061000, China.; 3 Department of Oncology, Affiliated Renhe Hospital of China Three Gorges University, Yichang, Hubei, 443001, China.; 4 Key Laboratory of Endocrinology of National Health Commission, Department of Endocrinology, State Key Laboratory of Complex Severe and Rare Diseases, Peking Union Medical College Hospital, Chinese Academy of Medical Science and Peking Union Medical College, Beijing, 100730, China.; 5 Department of Oncology, Quyang First Hospital, Baoding, Hebei, 073100, China.; 6 Department of Oncology, Jinan Hospital of Integrated Chinese and Western Medicine, Jinan, Shandong, 271100, China.; 7 Department of Radiation Oncology, Nanyang Central Hospital, Nanyang, Henan, 473005, China.

**Keywords:** Prostatic neoplasms, Brachytherapy, High-dose-rate, Toxic effects, Clinical outcomes

## Abstract

**Objective:** Although single-fraction high-dose-rate brachytherapy (SFHDR-BT) for localized prostate cancer has been attempted in clinical trials, there is currently a lack of relevant medical evidence. It is essential to conduct a systematic analysis of the long-term safety and efficacy of SFHDR-BT.

**Materials and methods:** Comprehensive and systematic searches for eligible studies were performed in PubMed, Embase, and the Cochrane Library databases. The primary endpoints included safety and efficacy, represented by toxic effects, biochemical recurrence-free survival (bRFS) and overall survival (OS), respectively. The proportion rates were used as the effect measure for each study and were presented with corresponding 95% confidence intervals (CI).

**Results:** Eight studies met the inclusion criteria for quantitative analysis, including 552 patients. The median follow-up was 71.3 months (60-72.8 months). The estimates of cumulative occurrence for severe gastrointestinal (GI) and genitourinary (GU) toxic effects were 0 and 3% (95% CI 1-5%), respectively. The pooled cumulative incidence of grade ≥ 3 sexual dysfunction occurrence was 4% (95% CI 1-7%). The estimate of long term bRFS was 72% (95% CI 68-76%) and 90% (95% CI 85-95%) for long term OS.

**Conclusion:** In general, SFHDR-BT is well tolerated and associated with suboptimal clinical benefit in patients with localized prostate cancer. High-quality prospective studies of SFHDR-BT are necessary to verify its safety and efficacy.

## Introduction

The academic consensus acknowledges that enhancing the overall dosage of external beam radiation therapy can potentially lead to improved survival rates in patients with prostate cancer 1, 2. Brachytherapy, in comparison to external radiation therapy, offers the advantage of delivering a higher radiation dose. The National Comprehensive Cancer Network (NCCN) guidelines suggest brachytherapy as a suitable treatment for patients with low-risk and certain intermediate-risk localized prostate cancer. The advent of transrectal ultrasound in the 1980s revolutionized the field by providing a dependable method for the implantation of low dose rate (LDR) seeds and the administration of high dose rate brachytherapy (HDR-BT) 3, 4.

Data from the National Cancer Database (NCDB) reveal that seed implantation is more prevalently utilized and technologically advanced in clinical settings compared to HDR-BT 5. Although large-scale clinical randomized controlled trials on HDR-BT are sparse, preliminary studies have indicated potential improvements in the quality of life for patients suffering from urological cancers 6. Over recent decades, the evolution of HDR-BT has trended towards reduced fractionation and enhanced single-dose delivery 7. Presently, in technologically sophisticated medical facilities, the entire process of implantation and treatment under anesthesia can be efficiently completed within a two-hour timeframe. The expediency of HDR-BT brings it on par with LDR regarding practicality, convenience, and financial aspects, thereby prompting a reevaluation of HDR-BT's intrinsic benefits.

Clinical trial outcomes have established that in treating localized prostate cancer, multi-fraction HDR-BT as a sole therapy offers disease control on par with LDR brachytherapy and external beam radiotherapy 8, 9. However, the implementation of multi-fraction HDR-BT has revealed several drawbacks, including the necessity for frequent hospital stays, multiple implant procedures, and extended bed rest during treatment. These requirements, coupled with substantial resource utilization and logistical complexities, diminish its attractiveness, particularly when contrasted with the permanency of LDR seed implantation 10, 11. Additionally, the lower α/β ratio and heightened sensitivity of prostate cancer to hypo-fractionated radiotherapy have spurred growing interest in single-fraction HDR-BT (SFHDR-BT) 6. This method presents an advantage over LDR as it minimizes the need for repeated implants and offers benefits in terms of practicality, convenience, and toxicity profile 12.

Currently, SFHDR-BT is not commonly implemented in localized prostate cancer treatment, and there remains debate over the optimal dosing regimen and the selection criteria for patient risk groups. In addition, there are relatively few published studies on SFHDR-BT for prostate cancer. The majority of studies are single-center investigations, and there are limited prospective studies with a median follow-up time of more than 5 years. Hence, the objective of this recent systematic review and meta-analysis is to comprehensively assess the therapy's safety and effectiveness.

## Materials and methods

### Literature search

This meta-analysis adhered to the Preferred Reporting Items for Systematic Reviews and Meta-analyses (PRISMA) guidelines 13. Literature searches were conducted in Pubmed, Embase, and Web of Science up to December 2023. The following search terms or keywords were used: “prostate” AND “cancer OR neoplasms” AND “single OR one” And “dose OR fraction” AND “high dose rate OR HDR” AND “brachytherapy.” Additionally, abstracts from prominent academic conferences were reviewed. Furthermore, references in the selected studies were scrutinized to identify potentially relevant articles.

### Study selection

The inclusion criteria for the studies were as follows: 1) patients with localized prostate cancer who were primarily treated with HDR-BT radiotherapy; 2) prospective or retrospective randomized or non-randomized studies with a single group or multiple groups; 3) all patients in the treatment group received a single dose of HDR-BT (defined as more than 15Gy per fraction), with or without androgen deprivation therapy (ADT); 4) at least one major endpoint measure was reported, including gastrointestinal (GI) toxicity, genitourinary (GU) toxicity, long-term overall survival (OS), and long-term biochemical recurrence-free survival (bRFS); 5) a minimum follow-up duration extending beyond five years. Exclusion criteria included: 1) non-human experimental studies; 2) patients who received adjuvant radiotherapy or had metastatic prostate cancer or developed disease relapse; 3) non-English articles; 4) sample size fewer than 20.

### Quality assessment of publications

For the evaluation of methodological quality in non-randomized experimental studies, the methodological index for non-randomized studies (MINORS) tool was employed 14. In contrast, randomized controlled trials were assessed using the Jadad scale, which considers the generation of random sequences, the implementation of double-blind procedures, and the management of withdrawals and dropouts. Scores on this scale range up to a maximum of 5, where a score between 1 and 2 is indicative of poor quality, while scores from 3 to 5 reflect higher quality 15.

### Data extraction

Data extraction from the selected studies was systematically conducted by two researchers, L.X. and L.L.Y., following a pre-established protocol. Information gathered included the lead author's identity, publication year, follow-up duration, number of patients, administered radiation doses, levels of prostate specific antigen (PSA), TNM staging, evaluation criteria, and specifics of ADT, along with the respective outcomes. Discrepancies or disputes in data interpretation between the two primary investigators were reconciled through consultation with a third researcher, L.X.L.

### Outcomes

The primary endpoint of the study focused on safety, assessed through the incidence rates of GU, GI, and erectile dysfunction toxicities. Secondary outcomes emphasized efficacy, gauged by the rates of bRFS. The assessment of toxic events included both severe (grade ≥3) and grade 2 toxicities, with severe toxicity defined as events equal to or exceeding grade 3, largely in line with the Radiation Therapy Oncology Group (RTOG) or the Common Terminology Criteria Adverse Events (CTCAE) criteria. Events necessitating hospital admission or surgical intervention, or explicitly described as “severe,” were categorized as grade ≥3 toxicities. Studies not specifically identifying toxicities as either GU or GI were excluded. In instances of no toxic events, the analysis incorporated GU, GI, and erectile dysfunction effects at a rate of zero. Efficacy was measured through 5-6 years of OS and bRFS. Despite varied terminologies used in different articles for biochemical failure, such as bNED, bDFS, BCR, bPFS, and BFFS, all were unified under the Phoenix definition of biochemical failure (an increase of 2ng/mL or more above the PSA nadir) 16. Thus, these terms were considered interchangeable for bRFS evaluation.

### Statistical analysis

In this study, we quantified the incidence rates of specific events among patients. These rates were then depicted in forest plots, each accompanied by a 95% confidence interval (CI). To evaluate the consistency of these combined outcomes, we applied Cochran's Q test complemented by the Higgins I² statistic. The adoption of a random-effects model was deemed necessary when the I² statistic surpassed the 50% threshold and the P-value for heterogeneity fell below 0.1, signaling significant inconsistency among the results. Conversely, in scenarios where these criteria were not met, the fixed-effects model was employed. To gauge the impact of individual studies on the overall findings, sensitivity analyses were conducted. Furthermore, the potential for publication bias was investigated using Begg's funnel plot. The statistical processes were performed using the STATA 12.0 software suite (Stata Corp, College Station, TX, USA), and a P-value at or below 0.05 was considered indicative of statistical relevance.

## Results

### Literature search and summary of studies

**Figure 1:**
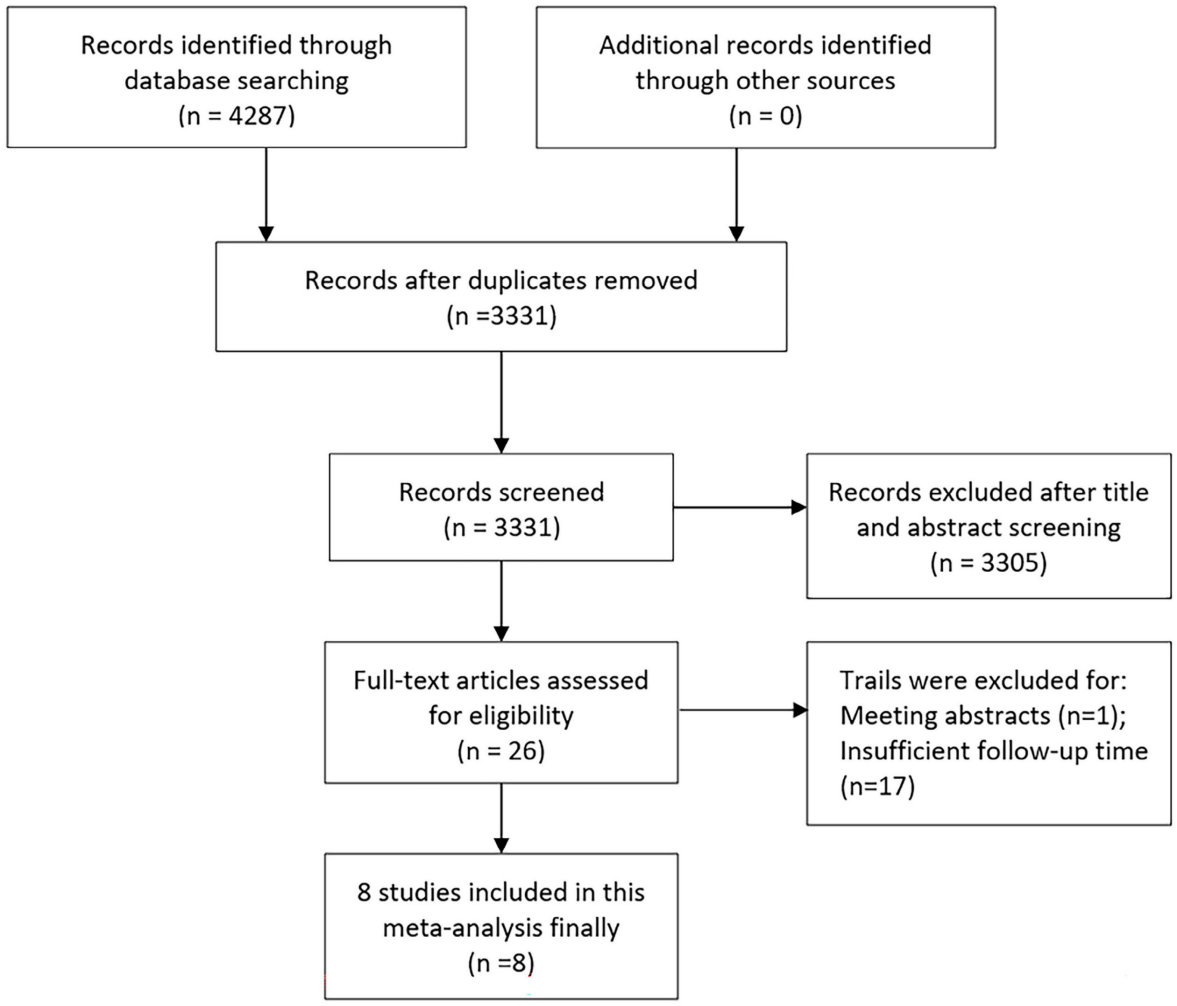
Flow chart of the included trials.

An initial search of relevant keywords resulted in identifying 4287 articles. Subsequent elimination of duplicate entries reduced this number to 3331. Further scrutiny of titles and abstracts led to the removal of 3305 articles. The next phase involved a detailed examination of the full texts and the integrity of the data, which led to the exclusion of 18 more studies. Ultimately, 8 articles met the eligibility criteria and were incorporated into the final meta-analysis 10, 11, 17-22. The process of article selection is illustrated in **Figure 1**. For assessing the quality of randomized controlled trials, the Jadad scale was employed, with studies scoring 3 or above being included. In contrast, non-randomized studies were appraised using the MINORS scale, requiring a score of 11 or more for inclusion (**Supplementary Table 1**).

A total of 552 patients were encompassed in the meta-analysis, with individual studies contributing between 33 and 87 patients each. Regarding the types of studies, there were more randomized controlled trials (five in total) compared to retrospective cohort studies (three in total), all published in the period from 2016 to 2023. **Table 1** displays the fundamental characteristics of these studies. The treatment regimens in these studies for SFHDR-BT in patients were inclusive of doses such as 19Gy, 20Gy, and 21Gy. The median duration of follow-up ranged from 60 to 72.8 months. Most studies 10, 11, 17-21 used CTCAE to assess toxic effects, and only one study 22 uses RTOG standards.

### Gastrointestinal toxicity

In the observed data, incidences of GI toxicity of grade 3 or higher were absent. Utilizing the weighted random-effects model, which indicated a significant heterogeneity (I² = 69.2%, P = 0.011), it was observed that the overall occurrence of grade 2 GI toxicity remained relatively low, being estimated at 9% with a 95% CI ranging from 4% to 14%.

### Genitourinary toxicity

**Figure 2:**
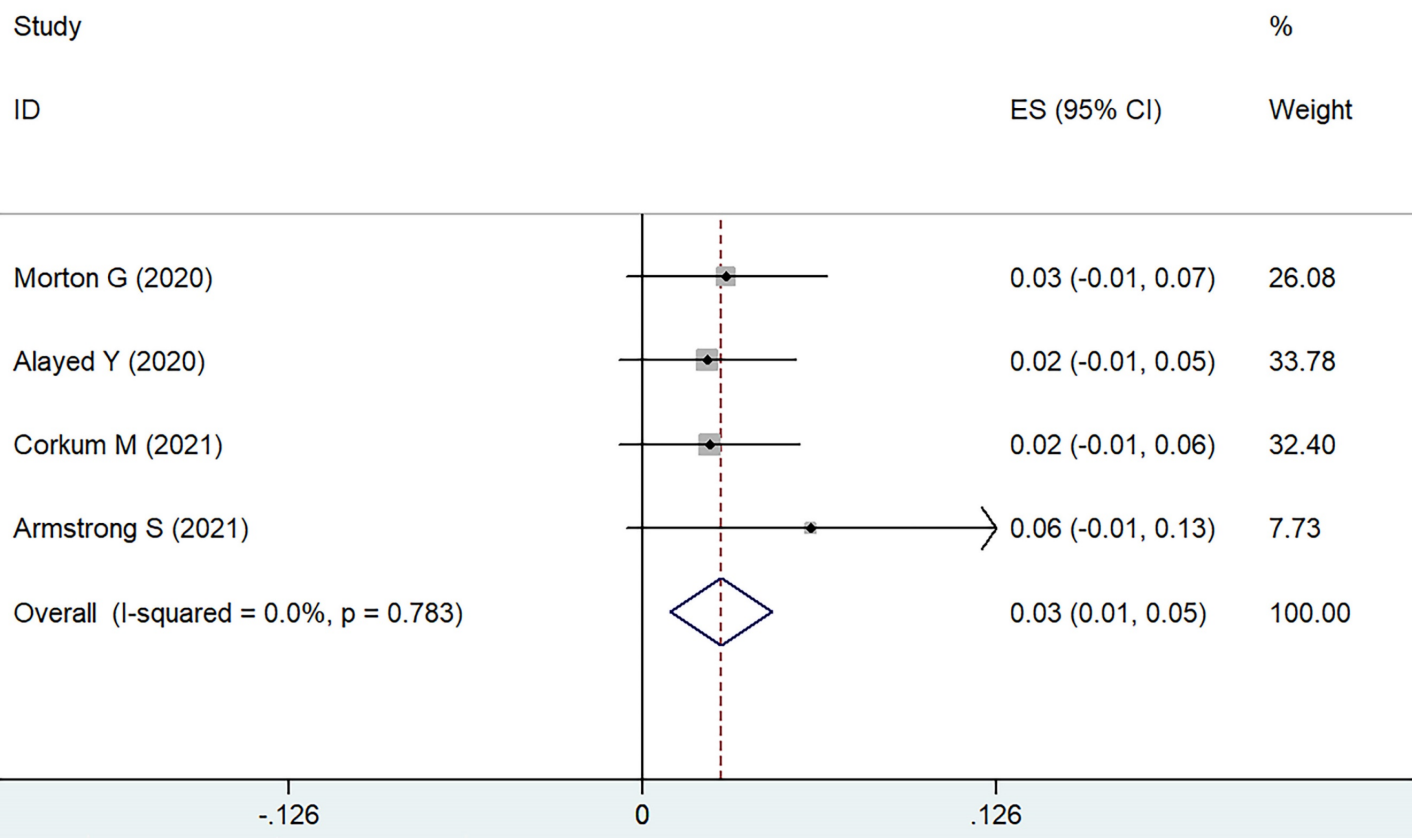
Forest plot for severe GU toxicity.

Occurrences of severe GU toxicity were uncommon, predominantly presenting as acute episodes of hematuria, occurrences of urethral stricture, and instances of urinary retention. Utilizing a weighted fixed-effects model, which demonstrated no heterogeneity (I² = 0, P = 0.783), the aggregated cumulative incidence was determined to be 3% (95% CI ranging from 1% to 5%; refer to **Figure 2**). Following SFHDR-BT treatment, the peak estimated incidence of grade 3-5 GU toxicity events was observed at 48 months.

### Sexual dysfunction

**Figure 3:**
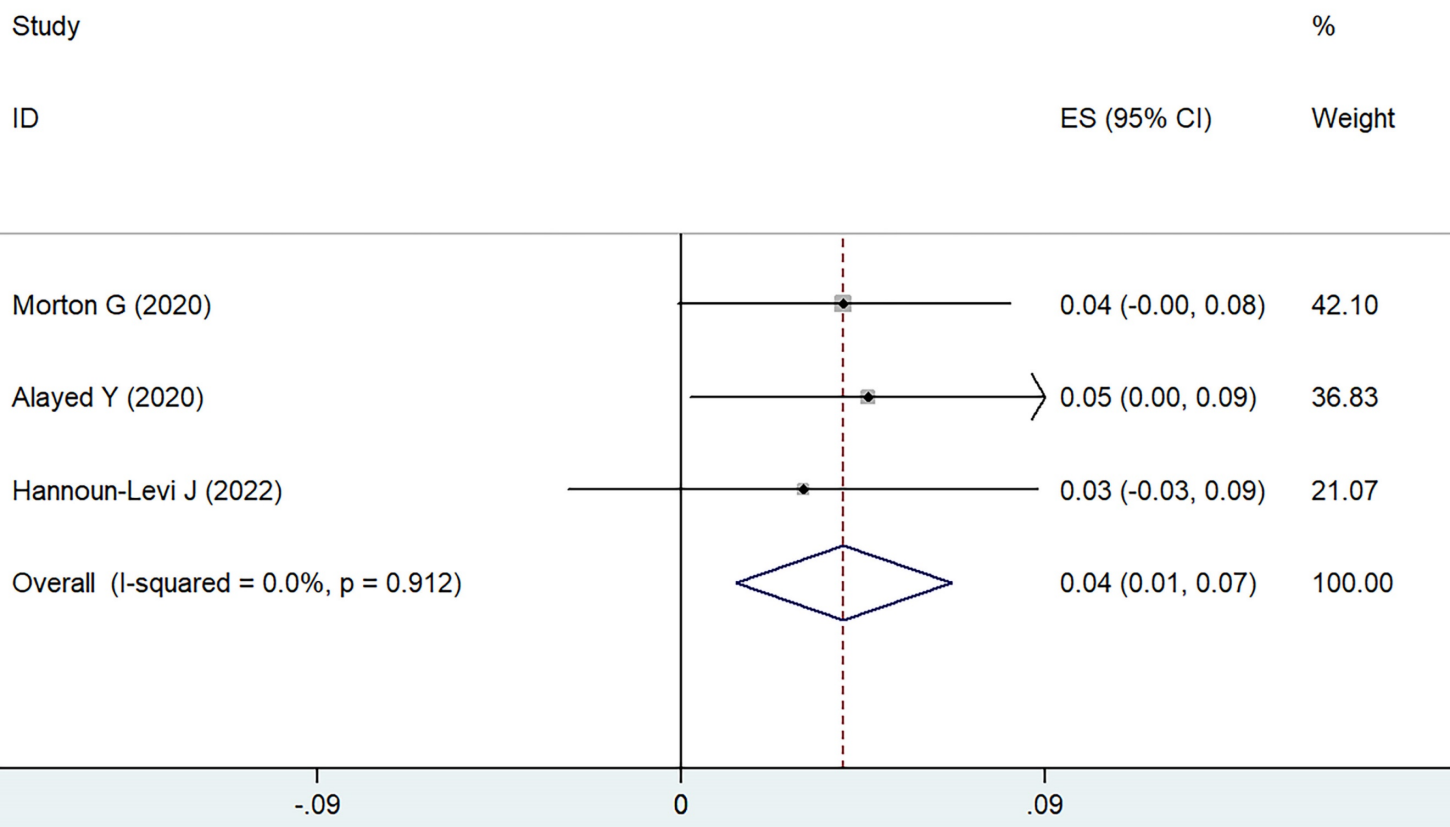
Forest plot for severe sexual dysfunction.

Utilizing the weighted fixed-effects model (I² = 0, P = 0.912), it was determined that the combined cumulative incidence of sexual dysfunction of grade 3 or higher was found to be 4%, with a 95% CI of 1-7%, as shown in **Figure 3**. In contrast, the cumulative incidence for grade 2 sexual dysfunction was notably higher, estimated at 30% (95% CI ranging from 17-43%). Erectile dysfunction is primarily observed as the main manifestation of sexual dysfunction.

### Long-term biochemical recurrence-free survival

**Figure 4:**
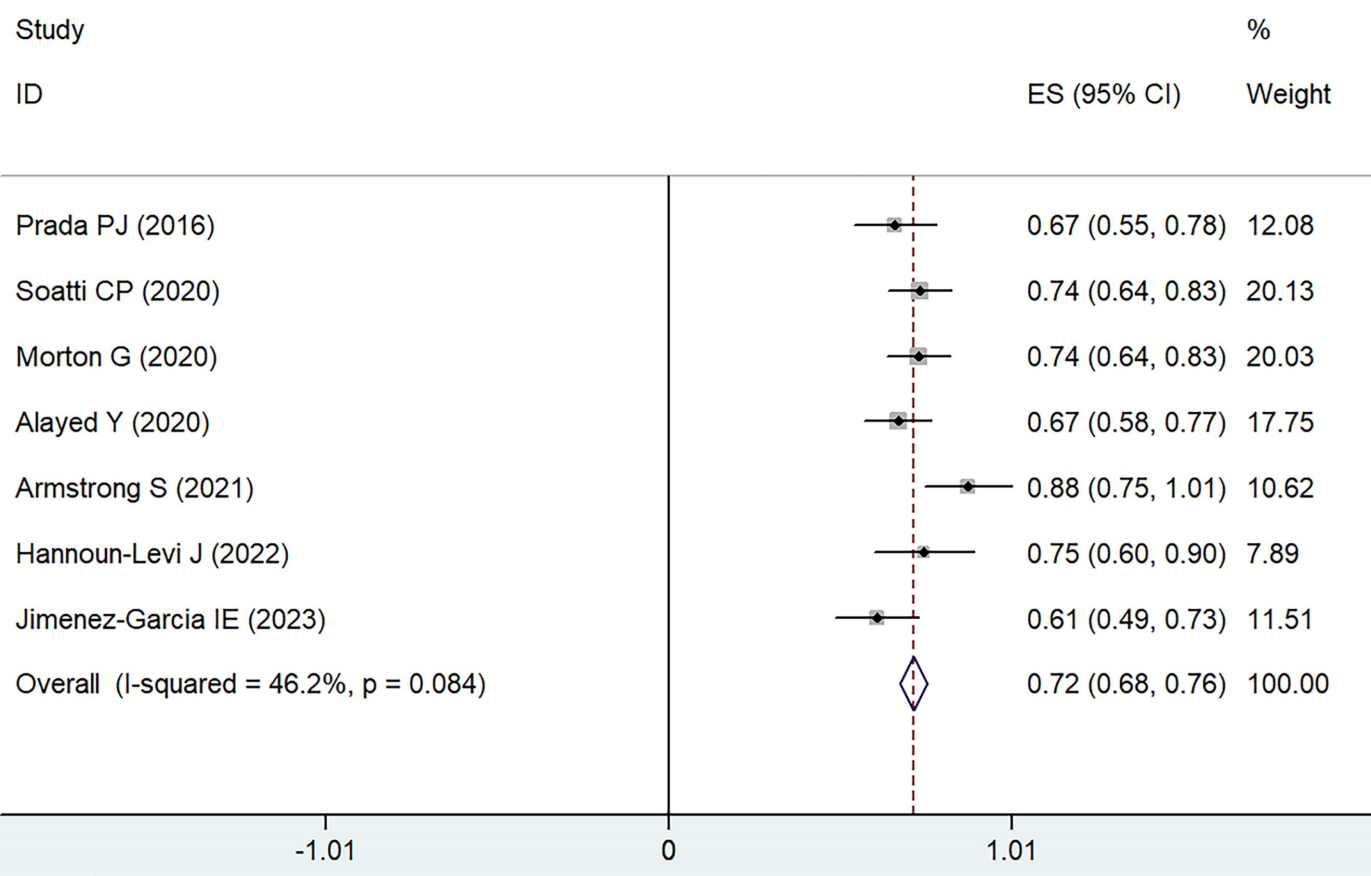
Forest plot for 5-6 years bRFS.

Data were gathered from eight different treatment groups across seven studies, showing a range in 5-6 years bRFS from 61.1% to 88%. Employing a weighted fixed-effects model, which indicated moderate heterogeneity (I² = 46.2%, P = 0.084), the combined rate of bRFS was calculated to be 72% with a 95% CI of 68-76% (as illustrated in **Figure 4**).

### Long-term overall survival

**Figure 5:**
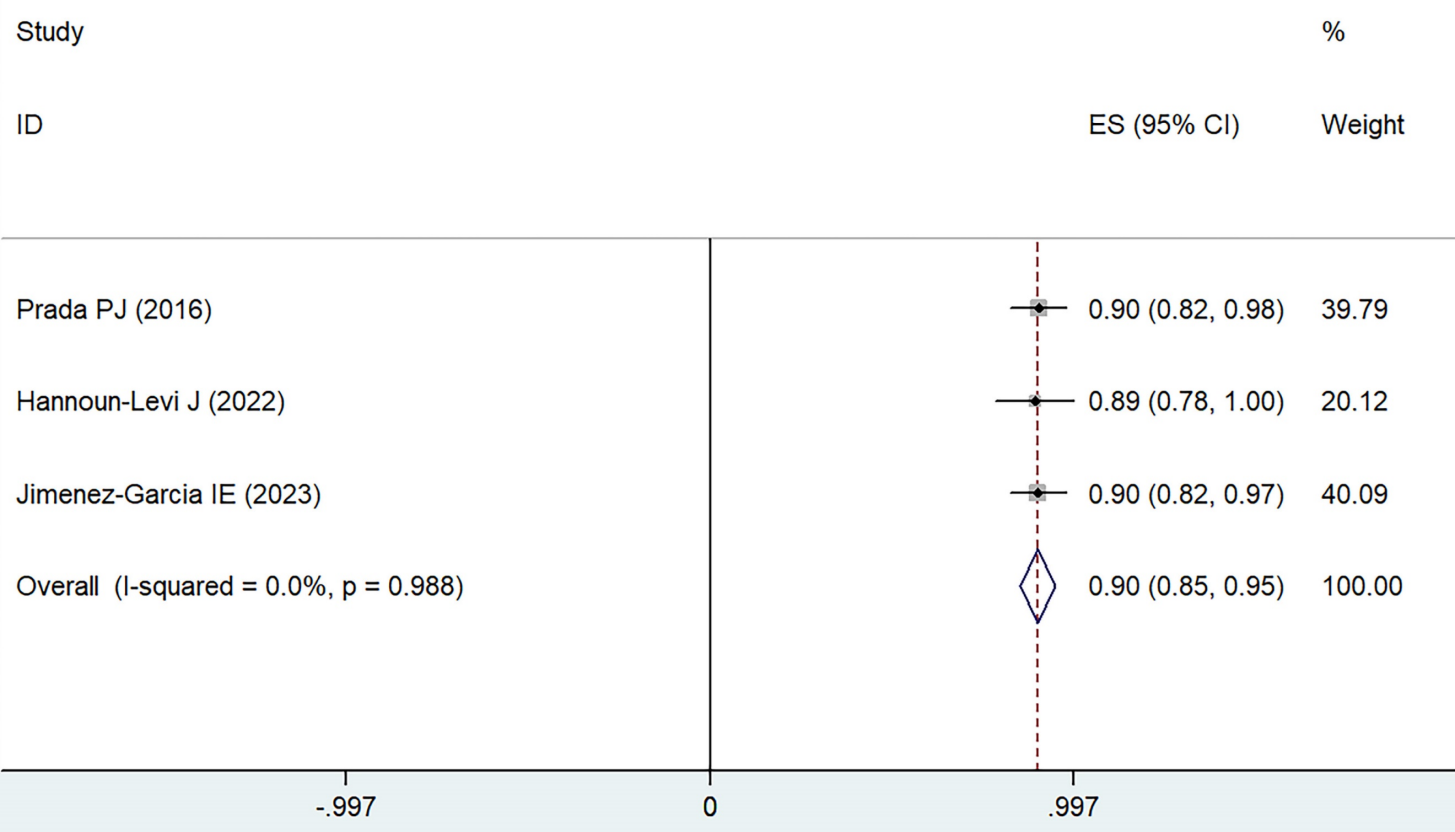
Forest plot for 5-6 years OS.

Data were extracted from eight different treatment groups across three studies, indicating that the OS rates for 5-6 years fluctuated between 89% and 90%. Employing a weighted fixed-effects model (I² = 0, P = 0.988), the aggregated rate was established at 90%, with a 95% CI of 85-95%, as depicted in **Figure 5**.

### Subgroup analysis

The results of subgroup analysis (based on sample size, PSA level, total dose and study design) are presented in **Table 2, 3**. None of the above factors affect bRFS and severe GU toxicity outcomes (P > 0.05).

### Sensitivity analysis and publication bias

**Figure 6:**
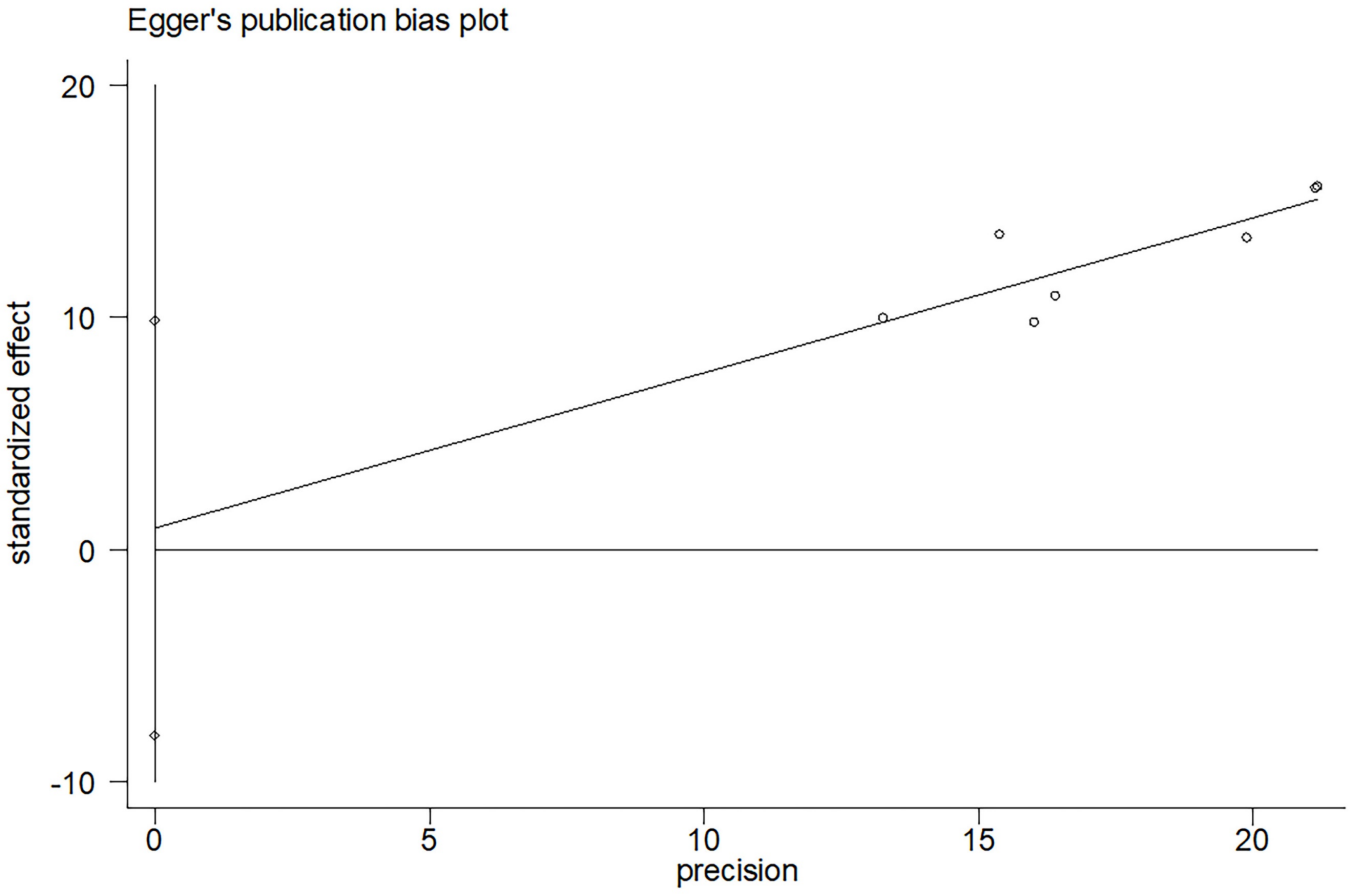
Funnel plots evaluating 5-6 years bRFS.

**Figure 7:**
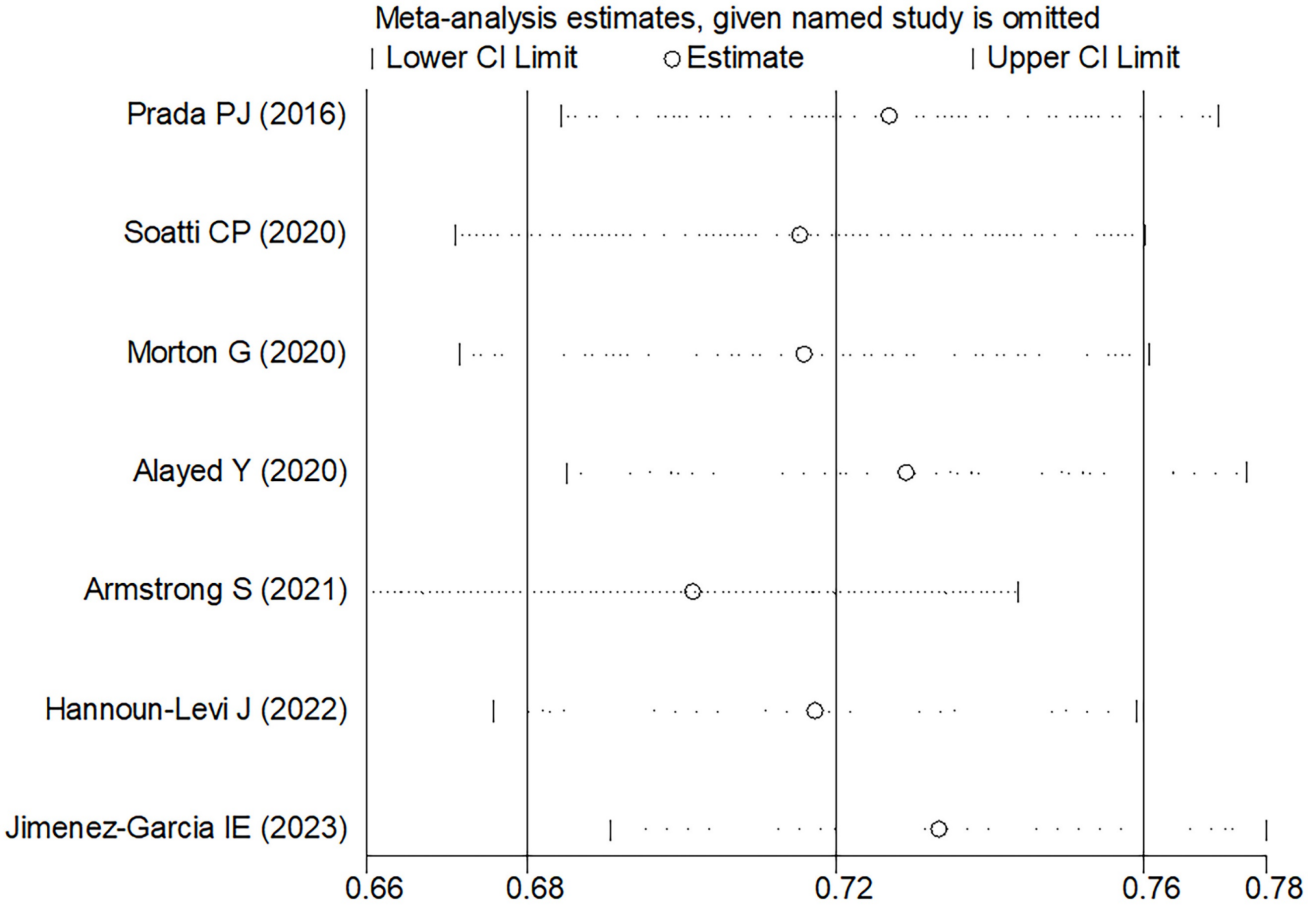
Sensitivity analysis of the 5-6 years bRFS.

In the comprehensive analysis of outcomes, the Egger's funnel plot analysis revealed no significant asymmetry, indicating no statistical bias (as depicted in **Figure 6**). To ascertain the reliability of the meta-analysis results, a sensitivity analysis was conducted. The results of this analysis confirmed the stability of the findings, demonstrating that the overall outcomes were not significantly influenced by any single study included in the analysis (refer to **Figure 7**).

## Discussion

The established safety and effectiveness of multi-fraction HDR-BT as a standalone treatment are well-documented, with research indicating both low levels of toxicity and high rates of bRFS 8, 23. Nonetheless, the complexities associated with multi-fraction treatments, including the use of resources, cost management, and patient convenience, have led to an increased focus on studies exploring SFHDR-BT in the context of localized prostate cancer. To the best of our knowledge, this meta-analysis represents the inaugural comprehensive long-term follow-up study examining the safety and efficacy of SFHDR-BT in treating localized prostate cancer.

Our study's results underscore the safety profile of SFHDR-BT, demonstrating minimal severe GI and GU toxicities with cumulative incidences at 0% and 3%, respectively. These outcomes suggest that a single dose of HDR-BT might offer toxicity profiles comparable to those of fractionated HDR-BT, LDR-BT, SBRT, and EBRT 22, 24-26. Research by Hoskin P *et al.* 27 revealed that multi-fraction HDR-BT regimens had a 6% incidence of grade 2 or higher GI and GU toxicities. A comparative study between SFHDR-BT and LDR-BT found no significant discrepancies in GI and GU toxicity levels 22. Significantly lower ≥ grade 2 GI toxicity rates were observed with SFHDR-BT compared to fractionated SBRT, as noted in a study (P < 0.05) 28. The CHHiP trial reported acute ≥ grade 2 bladder and bowel toxicities at 49% and 38% following hypofractionated radiotherapy 25. In contrast, our study noted crude estimates below 30% post-SFHDR-BT treatment. The standard arm of the FLAME trial reported late ≥ grade 2 GI and GU toxicities at 12% and 23% after EBRT, higher than those observed with SFHDR-BT 29. Our findings indicate that the toxicity profile of current SFHDR-BT dosages is well-tolerated, not significantly hindering the progression of these studies. It is important to note that severe toxic effects can evolve from lower-grade toxicities, emphasizing the need for early intervention and vigilant monitoring to mitigate the risk of increasing toxicity. While escalating the dose in a single fraction appears viable, careful consideration of the balance between toxicity and clinical benefit is imperative.

Current research on sexual function toxicity post-HDR-BT treatment for prostate cancer is limited, and studies focusing on SFHDR-BT are even scarcer. Our study's findings indicate that SFHDR-BT is generally well-tolerated, with a reported 4% incidence of severe sexual dysfunction. In contrast, the occurrence of grade 2 sexual dysfunction was notably more prevalent, estimated at 30%. Erectile dysfunction is predominantly the main form of sexual dysfunction observed. Viani GA *et al.* 30 documented a cumulative rate of late erectile dysfunction at grade 2 and grade ≥3 being 29.2% and 9%, respectively. Harris AA *et al.* 31 observed that the maximum rate of grade 2 physician-graded sexual toxicity was 53%, while grade 3 sexual toxicity was not reported. Furthermore, sexual scores relating to Health-Related Quality of Life did not revert to baseline levels even 18 months post-treatment. These studies suggest that while the incidence of grade 2 sexual function toxicity following HDR-BT is considerable and has an extended recovery period, instances of more severe sexual function toxicity are exceedingly rare.

In evaluating the effectiveness of single-dose HDR-BT, it was found to offer moderate biochemical control, with a 72% bRFS over 5-6 years. It is important to recognize that a 19Gy dose aligns well with the radiobiological principles, equating to a biologically equivalent dose (BED) comparable to 2×13Gy and 3×10.5Gy in HDR-BT for prostate cancer, considering an assumed α/β ratio of 1.4 32. A prospective study echoed these findings, showing nearly 90% 4-year bRFS with similar treatment protocols, although the difference was not statistically significant (P > 0.05) 27. It is widely acknowledged that the majority of biochemical recurrences in SFHDR-BT are localized failures, predominantly occurring at the initial disease site 19, 33. This pattern has steered researchers towards a novel therapeutic strategy: integrating a local boost with SFHDR-BT at the lesion site, although the clinical advantages of this approach have not been fully realized 10, 11. The resistance of tumor cells, especially cancer stem cells (CSCs), due to their complex biochemical mechanisms and proficient DNA repair capabilities, remains a pivotal concern, despite macro-level therapeutic effects 34. The lack of reoxygenation in SFHDR-BT might diminish tumor radiosensitivity 35. Additionally, tumor heterogeneity implies that cells with higher α/β ratios might be relatively resistant to single-fraction radiotherapy, hindering effective suppression or eradication of tumor cells 11. Furthermore, patient selection for SFHDR-BT demands careful consideration. In low-risk patients, 3-year bRFS was estimated at 99.0% and 5-year bRFS at 80.9%, with these outcomes showing significant differences in risk stratification by bRFS (P < 0.05) 36.

This study included all clinical studies with a median follow-up duration exceeding five years. Incorporating more long-term follow-up studies can effectively avoid related biases. Additionally, we evaluated long-term sexual function outcomes post-treatment, making this the first meta-analysis to assess changes in sexual function following SFHDR-BT for localized prostate cancer.

Despite the inherent constraints associated with meta-analyses, our research encountered several notable limitations. Initially, the inclusion of a limited number of retrospective studies in our meta-analysis might have introduced bias in data aggregation. To enhance the robustness of these results, there is a need for an increased volume of rigorously structured clinical trials and superior-quality prospective studies. Additionally, the predominance of Caucasian patients in our study's cohort necessitates cautious application of these findings across diverse ethnic groups.

In patients with localized prostate cancer, SFHDR-BT has been found to exhibit good tolerability and limited clinical advantages. To further substantiate its safety and effectiveness, there is a crucial need for ongoing and future well-structured prospective studies.

## Supplementary Material

Supplementary table.

## Figures and Tables

**Table 1:** Study characteristics.

Study	Reference	Year	n	Median follow-up (m)	Median PSA (range) (ng/ml)	TNM or risk group	Total dose	ADT(%/n)	Score Criteria	Outcomes
Prada PJ	17	2016	60	72	7 (4.40-15)	T1c-T2c Low to Intermediate Risk	19Gy	33/20	CTCAE v4.02	OS, bRFS, PFS, BC Acute GI, GU Late GI, GU
Soatti CP	18	2020	87	72	7.85 (1.8-59.5)	T1c-T2c All risk	19-20Gy	NR	CTCAE v5.0	bRFS
Morton G	19	2020	87	60	6.43 (4.71-8.90)	T1c-T2a Low to Intermediate Risk	19Gy	NR	CTCAE v4.0	bRFS Late GI, GU Erectile toxicity
Alayed Y	10	2020	87	62	6.42 (4.61-8.8)	T1c-T2c Low to Intermediate Risk	19Gy	None	CTCAE v4.0	BC, bRFS Late GI, GU Erectile toxicity
Corkum M	20	2021	87	63	6.43 (4.71-8.90)	T1c-T2a Low to Intermediate Risk	19Gy	None	CTCAE v4.0	Late GI, GU
Armstrong S	11	2021	50	70.6	23.8 (5.2-65.6)	T1c-T3a All risk	21Gy in a single fraction, two de-escalation prescription schedules based on V19Gy for PTV non-boost regions	NR	CTCAE v4.0	bRFS, BC Acute GI, GU Late GI, GU
Hannoun-Levi J	21	2022	33	72.8	8 (3.2-14.7)	T1c-T2a Low to Intermediate Risk	20Gy	None	CTCAE v4.0	OS, bRFS, DFS, CSS, BC Acute GI, GU Late GI, GU Erectile toxicity
Jimenez-Garcia IE	22	2023	61	72	7.19 (4.4-15.0)	T1-T2 Low to Intermediate Risk	19Gy	45.9/28	RTOG scales	OS, bRFS Late GI, GU

BC: Biochemical control; RTOG: Radiation Therapy Oncology Group; GI: gastrointestinal; GU: Genitourinary; NR: Not reported; ADT: Androgen deprivation therapy; CTCAE: Common Terminology Criteria of Adverse Events; OS: overall survival, bRFS: biochemical recurrence-free survival; TNM: Tumor-node- metastasis.

**Table 2:** Subgroup analysis of bRFS

Items		No. of studies	P value
Study design	Retrospective study	2	>0.05
	RCT	6	
Sample size	≤60	5	>0.05
	>60	3	
Median PSA	≤7ng/ml	3	>0.05
	>7ng/ml	5	
Total dose	≤19Gy	5	>0.05
	>19Gy	3	

bRFS: biochemical recurrence-free survival; PSA: prostate specific antigen; RCT: Randomized clinical trials

**Table 3:** Subgroup analysis of severe GU toxicity

Items		No. of studies	P value
Sample size	≤60	1	>0.05
	>60	3	
Median PSA	≤7ng/ml	3	>0.05
	>7ng/ml	1	
Total dose	≤19Gy	3	>0.05
	>19Gy	1	

PSA: prostate specific antigen; GU: genitourinary

## Abbreviations

NCCN: National Comprehensive Cancer Network
NCDB: National Cancer Database
HDR-BT: high dose rate brachytherapy
LDR-BT: low dose rate brachytherapy
SFHDR-BT: single-fraction HDR-BT
EBRT: external beam radiotherapy
GI: Gastrointestinal
GU: Genitourinary
ADT: androgen deprivation therapy
PRISMA: Preferred Reporting Items for Systematic Reviews and Meta-analyses guidelines
CI: Confidence interval
TNM: Tumor-node-metastasis
CTCAE: Common Terminology Criteria Adverse Events
RTOG: Radiation Therapy Oncology Group
MINORS: methodological index for non-randomized studies
PSA: prostate specific antigen
OS: overall survival
bRFS: biochemical recurrence-free survival
SBRT: stereotactic body radiotherapy
