# Supplementary material for: Single-fraction high-dose-rate brachytherapy as monotherapy for localized prostate cancer: long-term follow-up study based on meta-analysis
Source: J Cancer. 2025 Jan 1;16(2):533–42. doi: 10.7150/jca.104279 (PMC11685677; doi:10.7150/jca.104279)
Supplement: Supplementary file 1 — Supplementary table. [file jcav16p0533s1.pdf]

Supplementary Table 1. Risk of bias and quality assessment of studies included using MINORS (n=2)

Study	Methodological items for non-randomized studies								Additional criteria in the case of comparative study				Total scores
	A clearly stated aim	Inclusion of consecutive patients	Prospective collection of data	Endpoints appropriate to the aim of the study	Unbiased assessment of the study endpoint	Follow-up period appropriate to the aim of the study	Loss to follow up less than 5%	Prospective calculation of the study size	An adequate control group	Contemporary groups	Baseline equivalence of groups	Adequate statistical analyses	
Prada	2	2	2	2	0	2	2	2	-	-	-	-	14
Soatti	2	2	2	2	0	2	2	2	-	-	-	-	14
